# Host cell cAMP-Epac-Rap1b pathway inhibition by hawthorn extract as a potential target against *Trypanosoma cruzi* infection

**DOI:** 10.3389/fmicb.2023.1301862

**Published:** 2023-12-12

**Authors:** Gabriel Ferri, Lucía R. Fernández, Guillermo Di Mario, Daniel Musikant, Jorge A. Palermo, Martin M. Edreira

**Affiliations:** ^1^CONICET-Universidad de Buenos Aires, IQUIBICEN, Ciudad de Buenos Aires, Argentina; ^2^Laboratorio de Biología Molecular de Trypanosomas, Departamento de Química Biológica, Facultad de Ciencias Exactas y Naturales, Universidad de Buenos, Ciudad de Buenos Aires, Argentina; ^3^Departamento de Química Orgánica, Facultad de Ciencias Exactas y Naturales, Universidad de Buenos Aires, Buenos Aires, Argentina; ^4^Unidad de Microanálisis y Métodos Físicos Aplicados a la Química Orgánica (UMYMFOR), CONICET-Universidad de Buenos Aires, Buenos Aires, Argentina; ^5^Department of Pharmacology and Chemical Biology, School of Medicine, University of Pittsburgh, Pittsburgh, PA, United States

**Keywords:** *Trypanosoma cruzi*, cAMP signaling, EPAC, Rap1b, hawthorn, *Crataegus*, vitexin, host cell invasion

## Abstract

Although the two drugs currently available for the treatment of Chagas disease, Benznidazole and Nifurtimox, have proven to be effective in the acute phase of the disease, the 60–90-day treatment leads to high toxicity and unwanted side effects, presenting, in addition, a low efficacy in the chronic phase of the disease. For this reason, new therapies that are more effective are needed. In this regard, we have recently shown that the inhibition of the Epac-Rap1b pathway suppressed the cAMP-mediated host cell invasion by *Trypanosoma cruzi*. Interestingly, it has been described that vitexin, a natural flavone that protects against ischemia–reperfusion damage, acts by inhibiting the expression of Epac and Rap1 proteins. Vitexin can be found in plants of the genus *Crataegus* spp., traditionally known as hawthorn, which are of great interest considering their highly documented use as cardio-protectors. Pre-treating cells with an extract of *Crataegus oxyacantha* produced levels of *T. cruzi* invasion comparable to the ones observed for the commercially available Epac1-specific inhibitor, ESI-09. In addition, extract-treated cells exhibited a decrease in the activation of Rap1b, suggesting that the effects of the extract would be mediated by the inhibition of the cAMP-Epac-Rap1 signaling pathway. Using HPLC-HRMS^2^, we could confirm the presence of vitexin, and other flavones that could act as inhibitors of Epac/Rap1b, in the extracts of *C. oxyacantha.* Most significantly, when cells were treated with the extract of *C. oxyacantha* in conjunction with Nifurtimox, an increased modulation of invasion was observed.

## Introduction

Chagas disease (CD), also known as American trypanosomiasis, is a life-threatening illness caused by the flagellar protozoan *Trypanosoma cruzi*. Estimates indicate 7 million infected people and 75 million individuals are at the risk of contracting the disease, with an annual incidence of 30,000–40,000 cases and 10,000 deaths (WHO, 2020). Additionally, human migration from endemic areas to Europe, North America, and the Western Pacific Region significantly increased the seroprevalence of *T. cruzi* infection outside Latin America (Sangenito et al., 2020).

CD develops in an acute and a chronic phase (Echeverría et al., 2020). Chagas heart disease, a dilated cardiomyopathy, is the most common manifestation among the 30–40% of the patients who develop symptoms at the chronic stage of the disease (Nunes et al., 2018; Martinez et al., 2019).

There are currently two drugs available for the treatment of CD, Benznidazole (BNZ) and Nifurtimox (NFX) (Rodriques Coura and de Castro, 2002; WHO, 2020). However, since the efficacy of the highly toxic treatment decreases with the progression of the disease (Pérez-Molina and Molina, 2018), new targets and the development of new drugs that are more effective are required.

Signal transduction pathways, involved in *T. cruzi* life cycle, could offer multiple components to be targeted by new trypanocidal drugs (Pinto et al., 2014). Particularly, cAMP-mediated signaling plays a relevant role throughout the life cycle of *T. cruzi* (McDonough and Rodriguez, 2012) and could represent attention-grabbing molecular targets (Yan et al., 2016), considering the highly unusual effectors present in the parasite (Jäger et al., 2015) and that members of this pathway in the host cell were recently involved in invasion (Musikant et al., 2017; Ferri et al., 2023). In line with these observations, the cAMP pathway has been targeted in numerous human pathologies in the past. For example, the modulation of adenylyl cyclases (AC) activity by agonists/antagonists targeting G protein-coupled receptors (GPCRs) (Pavan et al., 2009; Pierre et al., 2009) or forskolin derivatives drugs (Toya et al., 1998; Alasbahi and Melzig, 2010) has been effectively utilized in the treatment of heart failure. Additionally, diseases such as chronic obstructive pulmonary disease, asthma, depression, schizophrenia, erectile dysfunction, psoriasis, and rheumatoid arthritis have been treated by modulation of phosphodiesterases (PDEs) (Lerner et al., 2000; Millar et al., 2005; Diamant and Spina, 2011; Page and Spina, 2011; Chiricozzi et al., 2016). In particular, specific inhibition of Epac (Exchange protein directly activated by cAMP) has shown a pharmacological effect on the invasion and metastasis of pancreatic and breast cancer, and protection against fatal rickettsiosis (Ahmed et al., 2019). Furthermore, Epac has been involved in a variety of physio-pathological mechanisms of cardiovascular disease (CVD), acting as a potential novel target for preventing and inhibiting the development of CVDs (Laudette et al., 2018; Tan et al., 2022). In this regard, we have previously shown that the activation of host’s Epac is the main mediator of cAMP-dependent invasion (Musikant et al., 2017) and, more recently, that this effect requires the activation and relocalization of the small GTPase of the Ras family, Rap1b (Ferri et al., 2023). Remarkably, our observations were confirmed in various cell lines, indicating that the activation of the cAMP/Epac/Rap1b pathway would be an ubiquitous mechanism of invasion of the host cell by the parasite (Ferri et al., 2023), making it an interesting target to explore further. With this in mind, we searched for natural compounds that could inhibit the pathway and which be used as an alternative antiparasitic agent. We found that vitexin, a flavonoid compound derived from natural products that protects against ischemia–perfusion (I-R) damage, may act by inhibiting the expression of Epac and Rap1 proteins, preventing mitochondria-mediated apoptosis (Yang et al., 2021). Vitexin is present in a wide variety of herbs traditionally used in alternative medicine. In particular, plants of the *Crataegus* genus, commonly known as Hawthorn, Thornapple, May-tree, Whitethorn, Mayflower or Hawberry, are of great interest due to their highly documented use as cardio-protectors (Jayalakshmi and Devaraj, 2010; Popovic-Milenkovic et al., 2014; Rastogi et al., 2016; Cuevas-Durán et al., 2017; Saoudi et al., 2019). It has been shown that pre-treatment with a *Crataegus* tincture prevented the damage induced by isoproterenol in rat hearts (Jayalakshmi and Devaraj, 2010). It is interesting to note that isoproterenol-induced myocardial infarction damage is correlated with an elevation of intracellular cAMP and the expression levels of Epac protein (Laurent et al., 2015; Surinkaew et al., 2019), leading to the presumption that it is an effect of the hawthorn extract on the pathway.

Considering the importance of the cAMP/Epac/Rap1b pathway in *T. cruzi* infection, we hypothesized that the inhibition of this pathway via natural cardio-protector compounds, such as the ones present in Hawthorn, could be used as an alternative and/or complementary treatment against CD. Therefore, we performed a screening in the medicinal herb databases searching for those approved for human use and which also have been proven to have protective effects against similar damages produced by the parasite in the heart. Finally, we analyzed the protective effects of the extracts and determined the mechanism of action and their potential use in combination with NFX.

## Materials and methods

### Cells and parasites

VERO (ATCC^®^ CCL-81™) and HELA (ATCC^®^ CCL-2™) cell lines were cultured in DMEM medium supplemented with Glutamax™ (Gibco), 10% (v/v) FBS (Natocor), 100 U/mL penicillin and 0.1 mg/mL streptomycin (Sigma) and maintained at 37°C in an atmosphere of 5% CO_2_. Tissue culture-derived trypomastigotes forms (TCT) of *T. cruzi* Y strain were routinely maintained in VERO cells cultured in DMEM supplemented with 4% FBS and penicillin/streptomycin. Trypomastigotes were obtained from the supernatants of infected VERO cells by centrifugation. First, the supernatant-conditioned medium was centrifuged at low speed (500 *g*) to remove intact cells and cell debris. Then, the supernatant obtained was centrifugated at 3,000 *g* for 15 min. and the pellet with the parasites was washed in PBS three times.

### *Crataegus oxyacantha* extracts

Assays were performed using the indicated dilution of a mother tincture (CO-EE) from aerial parts of *C. oxyacantha* (20% m/v hydroalcoholic extract), commercially available from Droguería & Herboristería Argentina (Timos SA). In a second strategy, a 1/10 filtered dilution of an infusion prepared with 4 g of dry aerial parts (commercially available from SoriaNatural^®^) in 100 mL of boiling water for 10 min (0.4% CO-Inf), was used to pre-treat cells.

### Host cell transfection

A transient transfection protocol with polyethyleneimine (PEI) was used (Longo et al., 2013). Briefly, cells were grown at about 60% confluence and incubated at 37°C in an environment of 5% CO_2_, 95% humidified air. The following day, cells were transfected with pCGN empty vector (EMPTY) or pCGN-HA-Rap1b (HA-Rap1) (kindly provided by Dr. D. Altschuler, University of Pittsburgh, United States) using a ratio of 4:1 PEI:DNA mix in OptiMEM medium (Gibco). The mixture was kept for 30 min at room temperature and then added to the cells and incubated at 37°C and 5% CO_2_. After 24 h, cells were washed with PBS and a complete medium (DMEM or Claycomb 10% FBS) was added. The transfected cells were used at 24 h post-transfection.

### Invasion assay

Cells were grown on glass cover slides in a 24 multi-well plate with DMEM, 10% FBS, for 24 h at 2 × 10^4^ cells/well density at 37°C, 5% CO_2_ and incubated with or without 37.5 μM of the Epac1-specific inhibitor ESI-09 (Sigma), dilutions of a CO-EE or a 0.4% CO-Inf DMSO was used as control for ESI-09 (DMSO Control) and the solvent where CO-EE was dissolved as control for CO-EE (solvent control). Cells were then washed and infected with trypomastigotes of the Y strain (moi 100:1) for 2 h. Parasites were removed and cells incubated for 48 h. In the case of cotreatment with NFX, the medium was changed to DMEM with the different concentrations of NFX or DMSO as control and incubated 48 h. Cells were fixed, stained with DAPI and the infection level determined by fluorescence microscopy. The percentage of invasion and amastigotes/100 cells were calculated counting 3,000 cells, expressed as mean ± SD of three or more independent experiments and performed in triplicate. Infection of non-treated cells was considered as a basal infection.

### Cytotoxicity assay

Cells were grown on glass cover slides in a 24 multi-well plate with DMEM 10% FBS for 24 h at 2 × 10^4^ cells/well density at 37°C, 5% CO_2_ and incubated with or without 37.5 μM of the Epac1-specific inhibitor ESI-09 (Sigma), 0.4% CO-EE or 0.4% CO-Inf cells were then washed and a solution of resazurin sodium salt was added as a fluorogenic oxidation–reduction indicator (final concentration 0.1 mM) (Rolón et al., 2006). After 3 h of incubation, fluorescence was measured with a FLUOstar OPTIMA (BMG LABTECH) microplate reader at 590 nm (excitation: 570 nm). Baseline-corrected values of fluorescence were normalized to the negative control. Results are expressed as mean ± SD of three or more independent experiments and performed in triplicate.

### Rap1b activation assays

HA-Rap1-transfected cells were incubated for 2 h with 0.04% of CO-EE or the control solvent (Ctrl). Detection of active Rap1b (GTP-bound) was performed through pull-down assays using a recombinant GST-RBD protein (GST fusion to the Rap1b-binding domain of the RalGDS protein, which only recognizes active Rap). A total of 1 mL of bacteria lysates containing GST or GST-RBD were mixed by rotation with 40 μL of 50% GSH-Sepharose at 4°C for 1 h. The beads were centrifuged at 800 *g* for 2 min at 4°C and washed with lysis buffer. Lysates from HA-Rap1 transfected cells pre-treated for 2 h with CO-EE or control solvent, were infected with trypomastigotes of the Y strain (Tp Y), or mock-infected were incubated with RBD-glutathione-agarose resin for 1 h at 4°C. Resin was washed and eluted with cracking buffer for WB analysis.

### Western blot

After electrophoresis, the gel was equilibrated in 25 mM Trizma base, 192 mM glycine and 20% v/v methanol pH 8.3. Then, proteins were transferred to previously hydrated with methanol PVDF membranes (Amersham™ Hybond, GE Healthcare) in a vertical tank (Mini-PROTEAN^®^ Tetra Cell, Bio-Rad). After transfer, membranes were blocked with 20 mM Tris–HCl, 500 mM NaCl, 0.05% Tween and 5% non-fat milk, pH 7.5, and incubated with anti-HA (Roche) antibody. After incubation, the membrane was washed and incubated with rabbit horseradish peroxidase (HRP)-IgG antibody (Santa Cruz Biotechnology), washed again, and then revealed using 0.88 mg/mL luminol, 0.066 mg/mL p-coumaric acid, 6 mM H_2_O_2_, 100 mM Tris–HCl, and pH 8.8 solution. Chemiluminescence was recorded with the C-DiGit scanner (LI-COR) and band intensity was quantified with ImageJ and ImageLab 6.1 (Bio-Rad) software.

### HPLC-MS^2^

LC/MS analyses were performed on a RRLC Agilent 1,200 using a Luna C18 column (3 μm, 2.0 × 100 mm; Phenomenex, Torrance, CA, United States). The mobile phase consisted of water containing 0.1% formic acid (A) and the solvent (B) was methanol. The flow rate was 0.3 mL/min, and the column temperature was set at 30°C. Linear gradient elution was performed as follows: 10–75% B (0–25 min), 75–100% B (25–26 min), and 100% B (26–44 min). A diode array was used as a detector coupled to a mass spectrometer. Triplicates of each sample were carried out.

Mass spectrometric analyses were performed using a Bruker MicrOTOF-Q II mass spectrometer (Bruker Daltonics, Billerica, MA, United States), equipped with an electrospray ion source. The instrument was operated at a capillary voltage of 4.5 kV with an endplate offset of 500 V, a dry temperature of 200°C using N_2_ as dry gas at 6.0 L/min, and a nebulizer pressure of 3.0 bar. Multipoint mass calibration was carried out using a sodium formate solution from *m*/*z* 50 to 1,200 in a negative ion mode. Data acquisition and processing were carried out using the software Bruker Compass Data Analysis version 4.0 supplied with the instrument.

### Identification of metabolites

Raw data (deposited at public MassIVE repository doi:10.25345/C50K26N42) was converted to ABF and pre-processed using the MS-DIAL freeware. ALL-GNPS library was used to search for matches between the features table obtained for the most intense ions and known metabolites. The results were manually cured comparing MS^2^ spectra of compounds already reported for the species.

### Docking

For ligand preparation, 3D structure of each compound identified in *C. oxyacantha* extract was retrieved from PubChem in a SDF format. The downloaded files were converted into PDB and then PDBQT format with OpenBabel and Autodock Tools, respectively. For receptor refinement, a human Epac2-Rap1b crystal complex was chosen (Epac_active; PDB ID: 4MGI). Rap1b chain coordinates were removed manually from the PDB file. Protein structure was downloaded in PDB format and minimized using FoldX software (Schymkowitz et al., 2005), with default parameters. Minimized Epac_active was then converted to PDBQT format with Autodock Tools. For docking simulation, Vinardo algorithm (Smina) (Quiroga and Villarreal, 2016) was used: PDBQT file of each compound was used as a ligand and minimized model of AF_Epac2 as the receptor, also in PDBQT file. The entire receptor was used as the autobox for each ligand, so blind-docking simulations were performed. All molecular visualizations were made with PyMol software.

### Statistical considerations

Statistical analysis was conducted with GraphPad Prism 8.0.1 software (GraphPad Software, Inc., San Diego, CA). Data are presented as the mean ± standard deviation (SD), and all experiments were repeated at least three times. Data were analyzed by two-way analysis of variance (ANOVA), and differences between groups were assessed with Tukey’s post-test (*p < 0.05, ***p < 0.01, ****p < 0.001). For the pull-down assays, bands intensity was quantified with ImageJ and ImageLab 6.1 (Bio-Rad) software and statistical comparisons were made using a Student’s *t*-test (***p < 0.001).

## Results

### Hawthorns as a natural source of potential antiparasitic compounds

**Figure 1:**
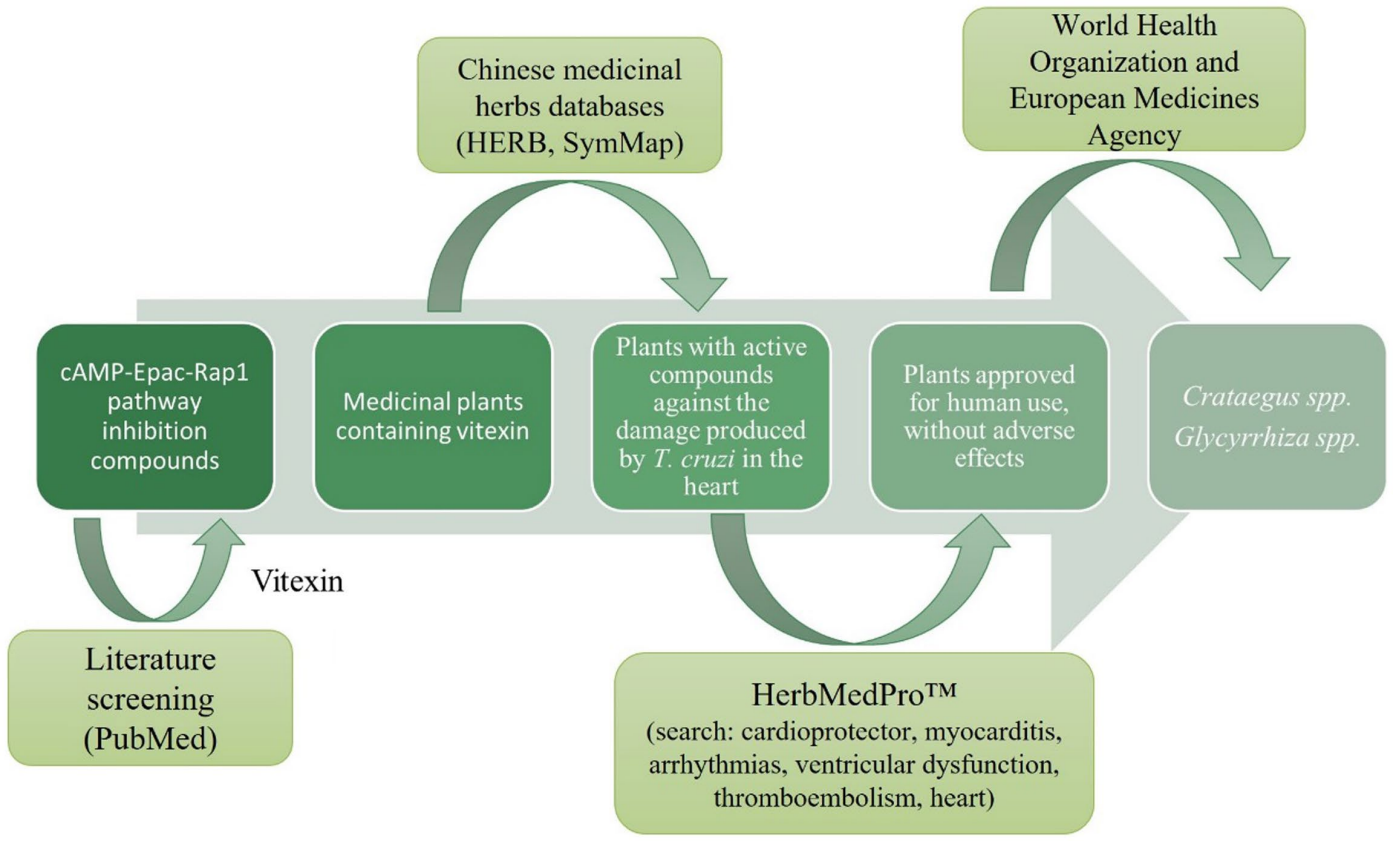
Screening of medicinal herbs that could potentially inhibit the cAMP/Epac/Rap1b pathway and protect against the damage caused by *T. cruzi* in the heart.

Taking into consideration that cAMP-dependent invasion of the host cell is mediated by activation in the host of the Epac1/Rap1b pathway (Musikant et al., 2017; Ferri et al., 2023), and that this pathway has already been targeted in numerous human disorders before, we hypothesized that aiming Epac/Rap1b would be an interesting strategy to suppress *T. cruzi* invasion as a potential new therapy against the parasite infection. Relatedly, there are reports in the literature describing that vitexin may act by inhibiting the Epac/Rap1 pathway (Che et al., 2016; Yang et al., 2021). Vitexin is a flavone present in a wide variety of herbs traditionally used in alternative medicine. By analyzing databases of Chinese medicinal herbs that contain vitexin (Wu et al., 2019; Fang et al., 2021), these data were verified with herbs approved for human use (World Health Organization, 1999; European Medicines Agency, 2016), which also present protective features against the damage caused by *T. cruzi* in the heart (American Botanical Council, 2023; Figure 1). For these reasons, *Crataegus* spp. plants, traditionally known as Hawthorns, would be great candidates as source of secondary metabolites with activity against *T. cruzi*.

### Hawthorn extracts inhibited cAMP-mediated invasion

**Figure 2:**
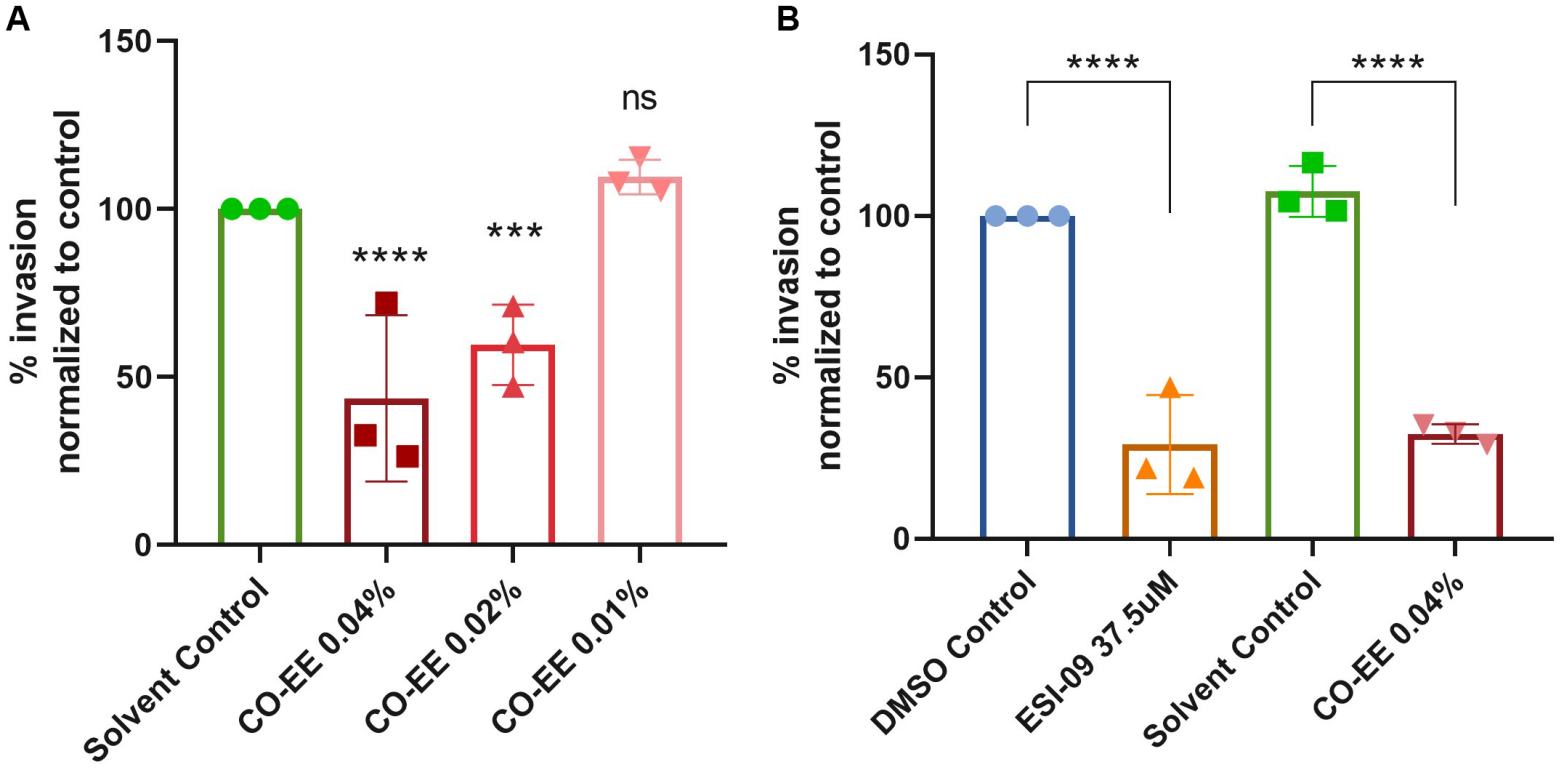
**(A)** Evaluation of CO-EE dose-dependent effect on *T. cruzi* invasion. HELA cells were pre-treated with 0.01, 0.02, and 0.04%, CO-EE for 1 h and then infected with trypomastigotes from *T. cruzi* Y strain (100:1 parasite to cell ratio for 2 h). After 48 h of infection, cells were fixed, stained with DAPI and percentage of invasion determined by fluorescence microscopy. **(B)** Comparison of the anti-parasitic activity of ESI-09 and CO-EE on *T. cruzi* invasion. HELA cells were pre-treated with 37.5 μM ESI-09 or 0.04% CO-EE for 1 h, and then infected with trypomastigotes from *T. cruzi* Y strain (100:1 parasite to cell ratio for 2 h). After 48 h of infection, cells were fixed, stained with DAPI and percentage of invasion determined by fluorescence microscopy. Infection of untreated cells was considered as basal infection. Results are expressed as mean ± SD (*n* ≥ 3). ****p < 0.0001, ***p < 00.1, two-way ANOVA and Tukey’s post-test.

**Figure 3:**
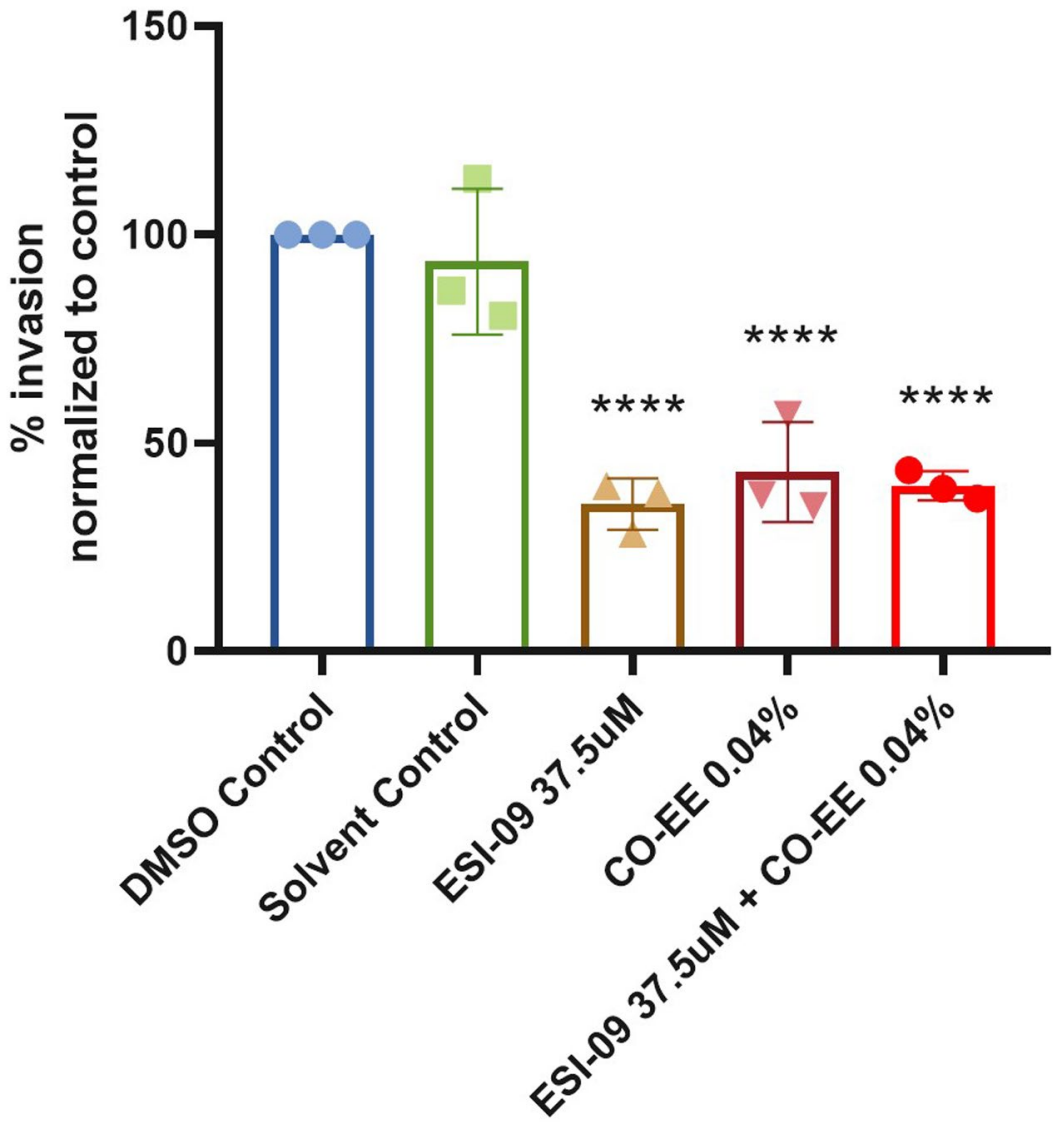
Modulatory effect of the CO-EE/ESI-09 cotreatment. Pre-treated HELA cells (37.5 μM ESI-09, 0.04% CO-EE or both for 1 h) were infected with trypomastigotes from *T. cruzi* Y strain (100:1 parasite to cell ratio for 2 h). After 48 h of infection, cells were fixed, stained with DAPI, and the percentage of invasion determined by fluorescence microscopy. Infection of untreated cells was considered as basal infection. Results are expressed as mean ± SD (*n* ≥ 3). ****p < 0.001, two-way ANOVA and Tukey’s post-test.

**Figure 4:**
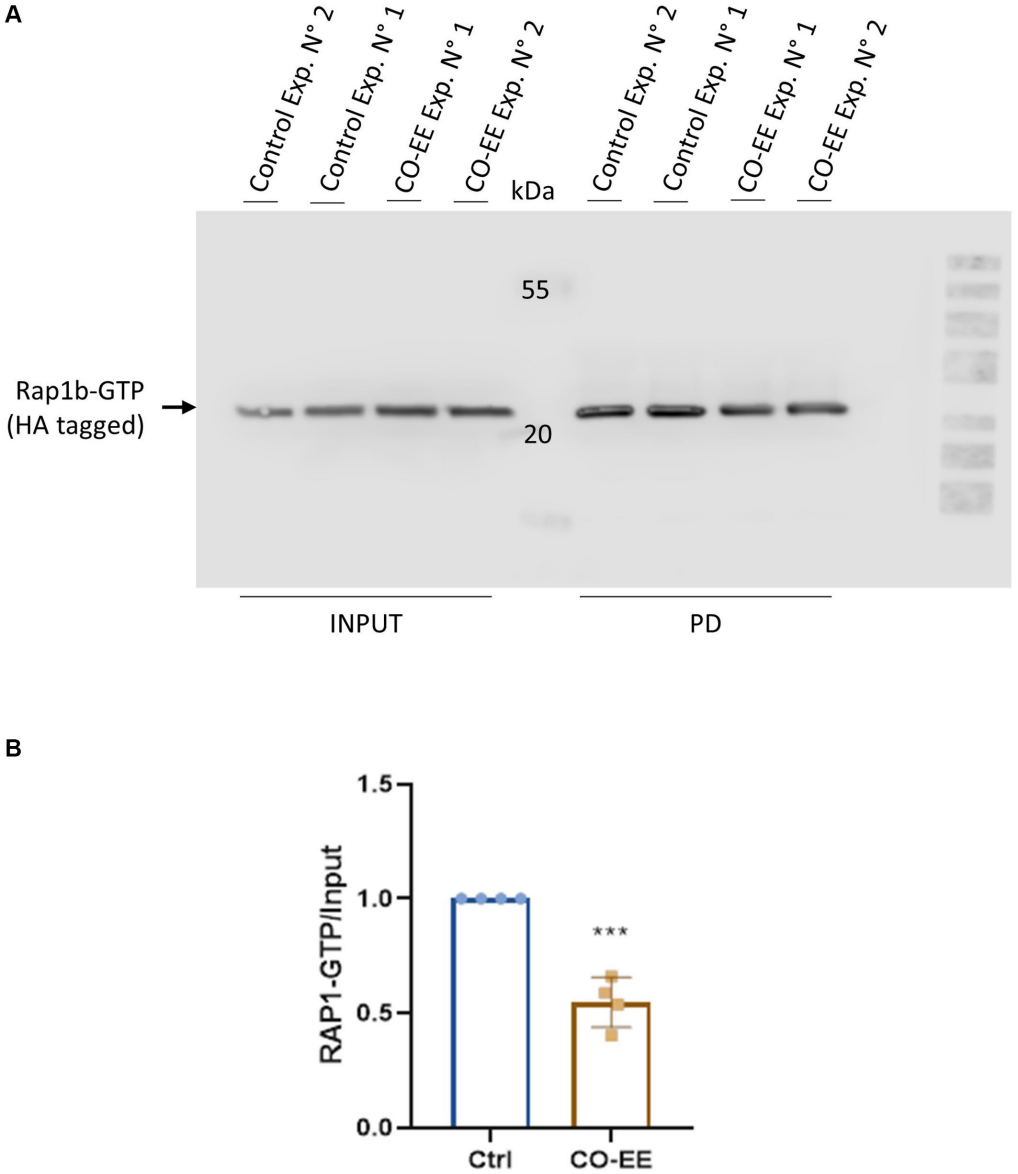
Rap1b activation assays. HA-Rap1b transfected HELA cells were incubated for 2 h with 0.04% of CO-EE. Cells were then lysed and pull-down assay with glutathione-agarose resin performed for 1 h at 4°C. The resin was washed and eluted with cracking buffer for WB analysis. **(A)** Representative WB of pull-down (PD) assay showing GTP-bound Rap1b of experiments 1 and 2. **(B)** Quantification of WB bands by densitometry. Bands were quantified and normalized against the INPUT using ImageJ and ImageLab 6.1 software (Bio-Rad). Results are expressed as mean ± SD (*n* ≥ 3). ***p < 0.001, Student’s *t*-test.

Invasion assays were performed in HELA cells that were pre-treated with different dilutions of a commercially available hydroalcoholic extract of *C. oxyacantha*, which was previously shown not to be toxic for cells (Supplementary Figure S1). The mother tincture produced a dose-dependent effect on *T. cruzi* invasion (Figure 2A), which was comparable to the modulation seen when using the Epac1-specific inhibitor (ESI-09) (Figure 2B). Interestingly, no additive or synergistic effects were observed when simultaneously pre-treating cells with ESI-09 and CO-EE, suggesting that the two inhibitors would share the same mechanism of action (Figure 3). To further support this hypothesis, we conducted pull-down experiments using lysates from cells incubated with CO-EE and the GST-tagged Rap1b-binding domain of RalGDS, a domain that exclusively recognizes the active GTP-bound form of Rap1b. These experiments revealed a reduction in Rap1b activation, strongly suggesting that CO-EE acts by inhibiting the Epac/Rap1b signaling pathway (Figure 4 and Supplementary Figure S2).

**Figure 5:**
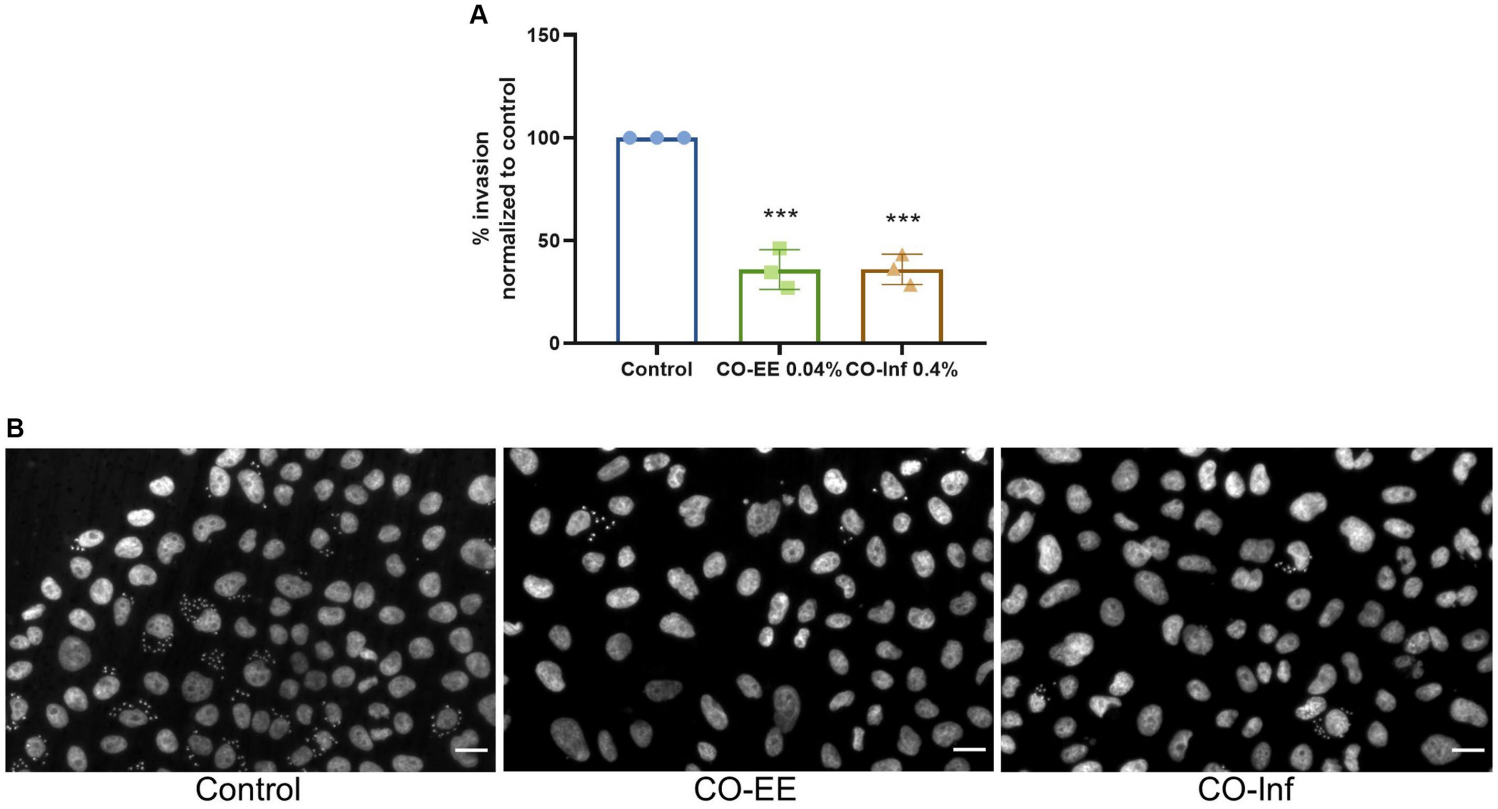
Evaluation of CO-inf modulatory action on *T. cruzi* invasion. **(A)** Pre-treated HELA cells (1 h at 0.04% CO-EE or 0.4% CO-Inf) were infected with trypomastigotes from *T. cruzi* Y strain (100:1 parasite to cell ratio for 2 h). After 48 h of infection, cells were fixed, stained with DAPI, and percentage of invasion determined by fluorescence microscopy. Infection of untreated cells was considered as basal infection. Results are expressed as mean ± SD (*n* ≥ 3). ***p < 0.001, two-way ANOVA and Tukey’s post-test. **(B)** Representative images of DAPI staining of infected cells pretreated with the indicated treatments.

In a second strategy to obtain secondary metabolites from *C. oxyacantha* with potential activity against *T. cruzi*, an infusion from dry leaves of this herb was prepared and tested in invasion assays. Noteworthy, cells pre-treated with this infusion showed a decrease in the levels of invasion, comparable to those obtained by pre-treating with CO-EE (Figure 5 and Supplementary Figure S3).

### Potential role of structurally related flavonoids in cAMP-mediated invasion inhibition

**Figure 6:**
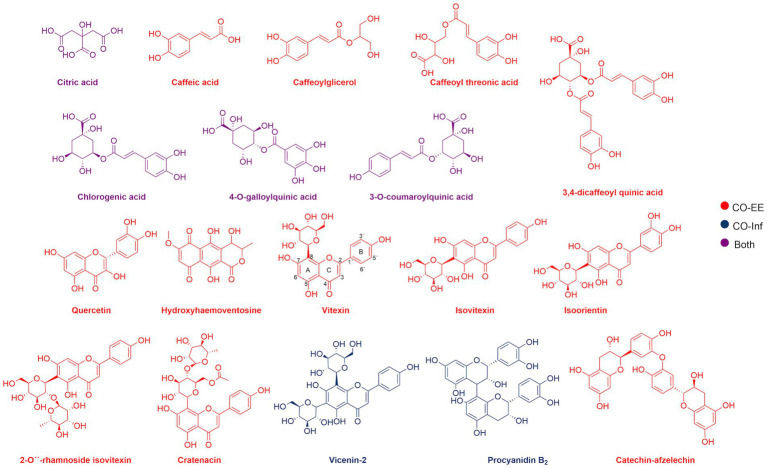
Chemical structure of compounds identified by LC/MS analyses in CO-EE (red), CO-Inf (blue), or both extracts (purple).

**Figure 7:**
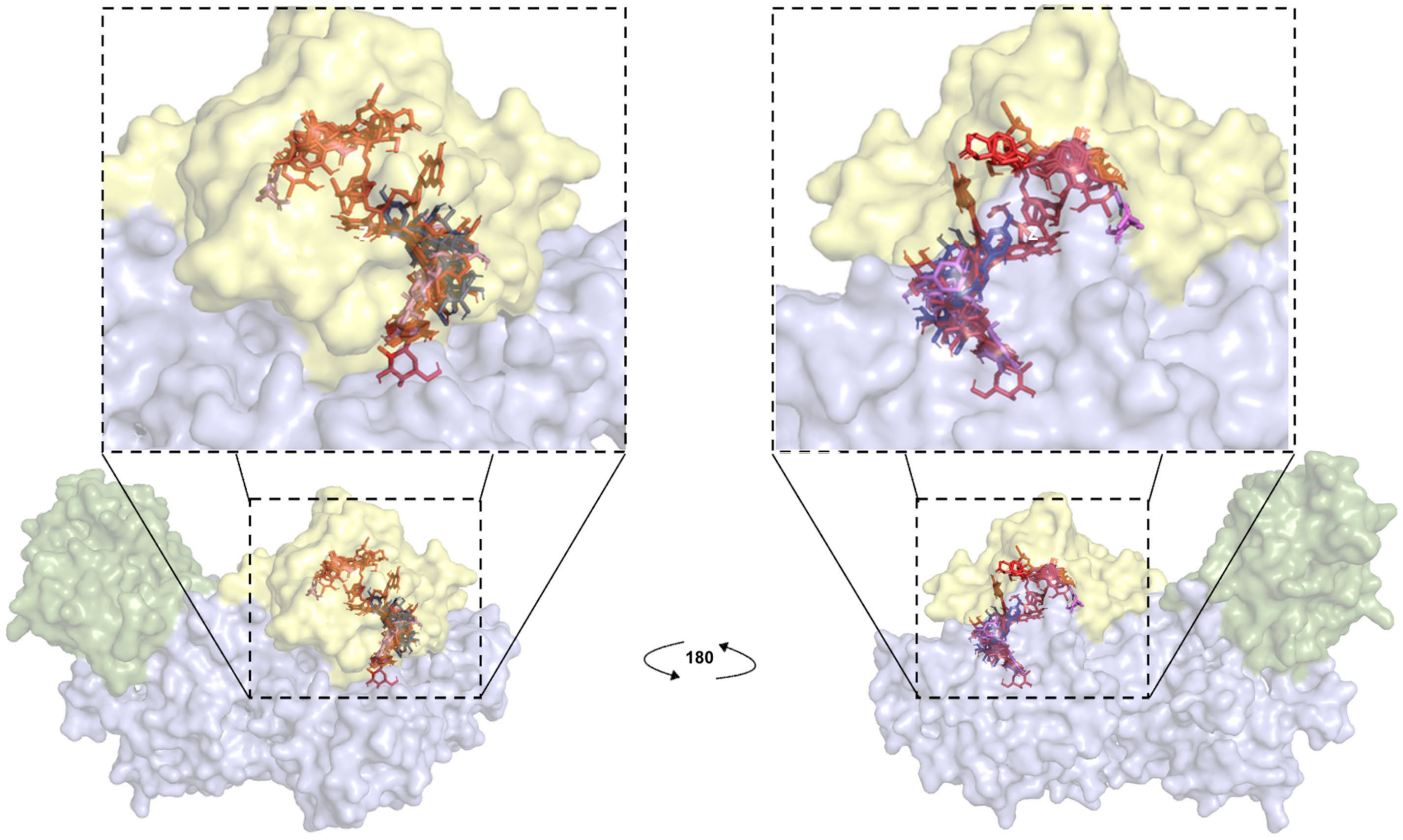
Blind docking simulations of flavonoid compounds present in *C. oxyacantha* extracts localized in Rap1b binding site of Epac. Each compound identified by LC/MS analyses in CO-EE and CO-Inf was used as a ligand for docking using a crystal structure of Epac2-Rap1b complex (PDB ID: 4MGI) as the receptor (Rap1b atoms were removed). All ligands, individually docked on Epac, are shown in the figure. Green: Epac regulatory region (CNBD + DEP; surface representation); blue: Epac catalytic region (REM + RA + GEF domains; surface representation). Rap1b original coordinates from crystal (yellow; surface representation) were then added to the final result of docking in order to show that the majority of these compounds were located in Epac/Rap1b interphase. Color for each ligand was assigned as in Figure 6.

**Figure 8:**
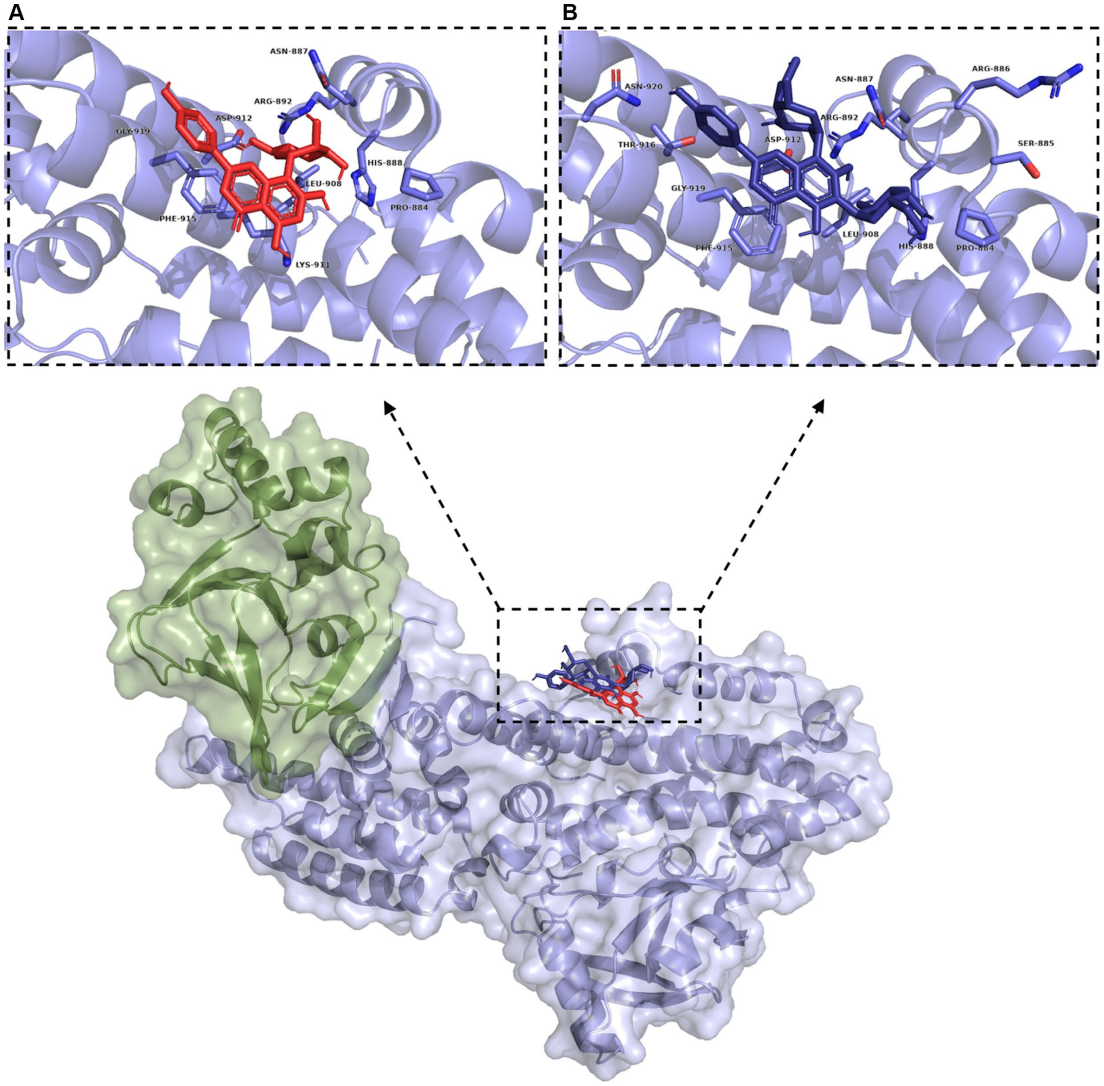
Blind docking simulations of vitexin (red) and vicenin 2 (dark blue) within the Rap1b binding domain of Epac (green: Epac regulatory region (CNBD + DEP; surface representation), blue: Epac catalytic region (REM + RA + GEF domains; surface representation)). Inset: Binding pockets for vitexin **(A)** and vicenin 2 **(B)**. Residues within 5 Å are labeled and shown in light blue.

LC/MS analysis confirmed the presence of vitexin in the CO-EE, along with several other additional flavonoids, already reported for the species (Table 1; Elsadig Karar and Kuhnert, 2016). Although vitexin was not identified in the CO-Inf, it that could be explained due to its poor solubility in water, but other related flavonoids were detected in this extract as well (Table 1; Zu et al., 2012). In addition to vitexin, blind docking experiments allowed us to identify 17 compounds (Figure 6; Table 2) that could also bind and potentially disrupt the Epac/Rap1b tridimensional interface (Figure 7), avoiding the activation of Rap1b and inhibiting the pathway. Noteworthy, apart from citric acid (which showed the lowest binding score), all other identified substances are polyphenols, ranging from simplest entities, like caffeic and coumaric acids and its esters, to more complex ones, such as dimeric flavonoids. Moreover, other related structures, such as isovitexin (Apigenin 6-C-glucoside) and 2′′-O-rhamnoside isoorientin, along with other flavanol dimers (Procyanidin B2 and Catechin-afzelechin) and esters of quinic and caffeic acids, were also predicted to bind in this domain with a similar, or even greater binding score, than vitexin (Table 2). Interestingly, among the identified glycosylated flavonoids, vicenin-2 (apigenin 6,8-di-C-glucoside), present in CO-Inf but absent in CO-EE, was predicted to bind with similar binding score and in the same pocket within Epac/Rap1b interaction domain than vitexin (Figure 8). These observations could explain why the CO-Inf is still active against *T. cruzi* infection even in the absence of vitexin.

**Table 1:** HPLC retention times (*t*_R_) and MS^2^ fragmentation in negative ion mode of CO-EE and CO-Inf (where NI, not identified; P, present; −, absent; NF, no fragments).

CO-Inf	CO-EE	*t*_R_ (min.)	Compound Identity	Mol. Form.	Theo. *m*/*z* [M-H]^−^	Exp. *m*/*z* [M-H]	Err. [ppm]	MS^2^ fragments (relative abundance %)
P	P	2.18	NI			273.0525		130 (100)
P	P	2.40	Sorbitol	C_6_H_14_O_6_	181.0718	181.0725	−3.9	NF
P	P	2.41	Glucopyranosyl glucitol	C_12_H_24_O_11_	343.1246	343.1250	−1.2	181 (100)
P	P	2.46	Gluconic acid	C_6_H_12_O_7_	195.0510	195.0508	1.0	163 (100)
P	P	2.50	4-*O*-galloylquinic acid	C_14_H_16_O_10_	343.0671	343.0635	10.5	191 (100)
P	P	2.53	Quinic acid	C_7_H_12_O_6_	191.0561	191.0560	0.5	191 (100), 173 (12), 179 (16), 153 (10), 127 (14)
P	P	3.11	Citric acid	C_6_H_8_O_7_	191.0197	191.0198	−0.5	NF
P	P	8.09	Caffeic acid (isomer)	C_9_H_8_O_4_	179.0350	179.0340	5.6	NF
P	P	8.11	Chlorogenic acid (Caffeoylquinic acid)	C_16_H_18_O_9_	353.0878	353.0857	5.9	191 (100), 179 (44)
-	P	9.90	Hydroxyhaemoventosine	C_15_H_12_O_8_	319.0459	319.0426	10.3	NF
-	P	9.92	Caffeic acid and sorbitol ester	C_15_H_20_O_9_	343.1035	343.1051	−4.7	181 (41), 179 (60), 161 (100), 135 (47)
-	P	10.09	NI			359.0790		NF
P	P	10.14	Coumaric acid	C_9_H_8_O_3_	163.0401	163.0410	−5.5	NF
P	P	10.14	Coumaroylquinic acid	C_16_H_18_O_8_	337.0929	337.0943	−4.2	163 (100), 119 (38)
P	P	10.98	NI			425.1387		NF
P	P	11.02	Procyanidine type B	C_30_H_26_H_12_	577.1351	577.1361	−1.7	425 (32), 407 (92), 289 (87)
P	-	11.10	NI			599.1802		NF
P	P	11.18	Chlorogenic acid (isomer)	C_16_H_18_O_9_	353.0878	353.0883	−1.4	191 (100)
-	P	11.90	Chlorogenic acid (isomer)	C_16_H_18_O_9_	353.0878	353.0885	−2.0	NF
-	P	11.91	Shikimic acid	C_7_H_10_O_5_	173.0455	173.0448	4.0	NF
-	P	11.95	Caffeic acid	C_9_H_8_O_4_	179.0350	179.0344	3.4	135 (100), 134 (37)
P	-	12.32	Procyanidine type C	C_45_H_38_O_18_	865.1985	865.1987	−0.2	865 (100), 713 (31), 577(37), 575 (37), 425 (26)
P	-	12.50	NI			389.1089		NF
-	P	12.65	(Epi)catechin-(Epi)afzelechin	C_30_H_26_O_11_	561.1402	561.1389	2.3	435 (14), 407 (10), 289 (100), 245 (24)
-	P	12.70	Caffeoylglicerol	C_12_H_14_O_6_	253.0718	253.0747	−11.5	161 (100)
P	P	13.17	(Epi)catechin	C_15_H_14_O_6_	289.0718	289.0715	1.0	245 (20), 221 (87), 203 (100), 151 (64), 123 (57)
P	P	13.17	NI			245.0829		NF
-	P	13.27	NI			327.1082		NF
-	P	13.11	Caffeoyl threonic acid	C_13_H_13_O_8_	297.0616	297.0608	2.7	179 (26), 135 (100)
-	P	13.74	NI			173.0585		NF
-	p	13.76	Coumaroylquinic acid (isomer)	C_16_H_18_O_8_	337.0929	337.0936	−2.1	173 (100), 163 (21)
P	-	15.57	Cyanidin	C_15_H_12_O_6_^+^	287.0550	287.0552	−0.7	NF
P	-	15.57	Cyanidin 3-*O*-hexoside	C_21_H_22_O_11_^+^	449.1089	449.1062	6.0	287 (100), 175 (9), 151 (57), 135 (9)
-	P	15.80	NI			593.2035		NF
-	P	15.91	Orientin (Luteolin-8-C-glucoside)	C_21_H_20_O_11_	447.0933	447.0943	−2.2	327 (100), 357 (68), 333 (29), 297 (16), 285 (15)
-	P	16.10	Isoorientin (Luteolin-6-C-glucoside)	C_21_H_20_O_11_	447.0933	447.0927	1.3	357 (100), 327 (72), 363 (65), 297 (15), 339 (13), 285 (13), 449 (12)
-	P	16.20	(Iso)orientin- 2”-*O*-rhamnoside	C_27_H_30_O_15_	593.1512	593.1485	4.6	593 (100), 473 (31), 327 (7), 429 (5)
-	P	16.55	Vitexin (Apigenin 8-C-glucoside)	C_21_H_20_O_10_	431.0984	431.0967	3.9	311 (100), 283 (18), 341 (14)
P	-	16.60	Vicenin-2 (Apigenin 6,8-di-C-glucoside)	C_27_H_29_O_15_	593.1512	593.1479	5.6	413 (100), 293 (41)
P	P	16.72	Vitexin-2”-*O*-rhamnoside	C_27_H_30_O_14_	577.1563	577.1542	3.6	577 (51), 413 (100), 293 (24)
-	P	17.27	Isovitexin (Apigenin 6-C-glucoside)	C_21_H_20_O_10_	431.0984	431.0984	0.0	311 (100),341 (52),283 (9),295 (9), 323 (9), 313 (7), 431 (6), 353(5)
-	P	17.44	Isovitexin-2”-*O*-rhamnoside	C_27_H_30_O_14_	577.1563	577.1593	−5.2	577 (100), 413 (61), 293 (35), 457 (13), 323 (5)
P	P	17.93	Hyperoside (Quercetin 3-*O*-galactoside)	C_21_H_20_O_12_	463.0882	463.0866	3.5	300 (100), 301 (34), 463 (12)
P	-	18.01	Rutin (Quercetin 3-*O*-(6-*O*-rhamnosyl-glucoside))	C_27_H_30_O_16_	609.1461	609.1471	−1.6	609 (100), 301 (15)
P	P	18.15	Quercetin 3-*O*-hexoside	C_21_H_20_O_12_	463.0882	463.0856	5.6	300 (100), 301 (42), 463 (10)
-	P	19.22	3,4-Di caffeoyl quinic acid	C_25_H_24_O_12_	515.1195	515.1200	−1.0	353 (100), 173 (65), 179 (38), 191 (15)
-	P	19.22	Isorhamnetin-*O*-hexoside	C_22_H_24_O_12_	477.1038	477.1059	−4.4	314 (100), 315 (77), 300 (64), 299 (50), 477 (41)
-	P	19.30	Quercetin *O*-hexoside (isomer)	C_21_H_20_O_12_	463.0882	463.0870	2.6	301 (100), 179 (14)
-	P	19.57	Cratenacin	C_29_H_32_O_15_	619.1668	619.1627	6.6	619 (59), 413 (100), 293 (39)
-	P	20.64	Ethyl caffeate	C_11_H_12_O_4_	207.0663	207.0648	7.2	179 (12), 161 (47), 135 (73), 134 (32), 133 (100)
-	P	21.50	Quercetin	C_15_H_10_O_7_	301.0354	301.0335	6.3	301 (12), 273 (6), 245 (7), 179 (30), 151 (100), 121 (7)
-	P	23.16	*O*-Methylquercetin	C_16_H_12_O_7_	315.0510	315.0510	0.0	300 (100), 255 (13), 216 (15)

**Table 2:** Blind docking scores of CO-EE compounds that are located in Epac/Rap1b interphase.

**Compound**	**Smina score (kcal/mol)**
4-*O*-galloylquinic acid	−5.2
Citric acid	−4.1
Caffeic acid	−5.5
Chlorogenic acid	−5.9
Hydroxyhaemoventosine	−5.1
3-*O*-coumaroylquinic acid	−5.7
Procyanidine B2	−5.8
Catechin-afzelechin	−7.5
Caffeoylglycerol	−7.1
Caffeoyl threonic acid	−4.9
Isoorientin	−6.1
Isovitexin	−6.9
2”-*O*-rhamnoside isoorientin	−7.8
Vitexin	−7.0
Vicenin-2	−6.8
3,4-di caffeoyl quinic acid	−7.1
Cratenacin	−5.6
Quercetin	−5.9

**Figure 9:**
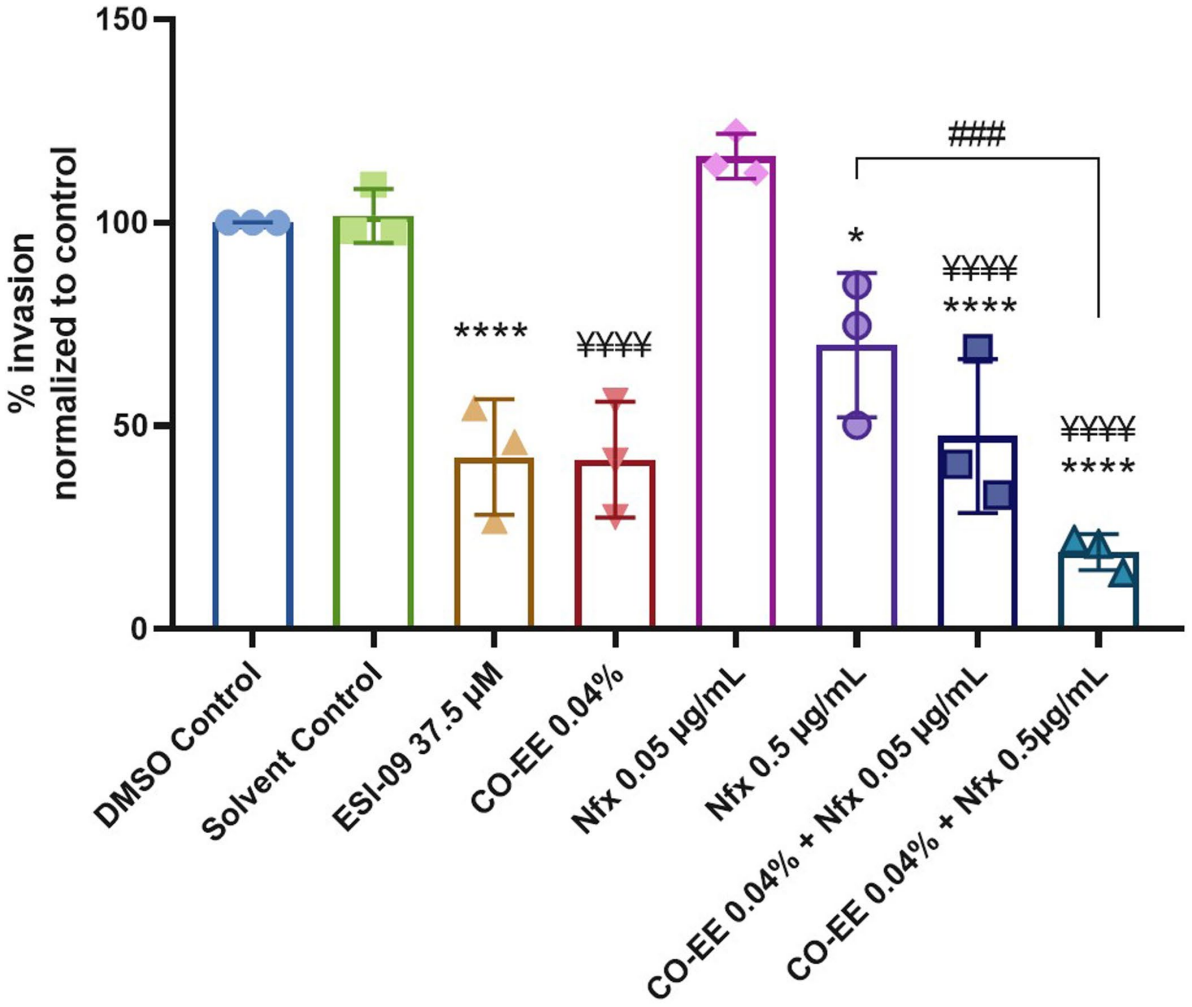
Inhibitory effect of the CO-EE/NFX cotreatment. Pretreated HELA cells (1 h at 37.5 μM ESI-09 or 0.04% CO-EE) were infected with trypomastigotes from *T. cruzi* Y strain (100:1 parasite to cell ratio for 2 h). The medium was changed to DMEM medium with different concentrations of NFX or DMSO as control and incubated 48 h. Then cells were washed, fixed, stained with DAPI and percentage of invasion determined by fluorescence microscopy. Infection of untreated cells was considered as basal infection. Results are expressed as mean ± SD (*n* ≥ 3). ****p < 0.0001, ^¥¥¥¥^*p* < 0.0001, ^###^*p* < 0.001, *p < 0.05, two-way ANOVA, and Tukey’s post-test. The asterisks (*) show the significance with the DMSO control, while the yen sign (¥) show the significance with the solvent control.

An approach that is often employed to avoid or decrease drug toxicity is the reduction of the time of treatment or dosage of the drug. To test the possibility of reducing NFX concentration, a cotreatment with CO-EE was evaluated in *in vitro* invasion assays. As shown in Figure 9 and Supplementary Figure S4, the cotreatment with NFX/CO-EE showed to be significantly more effective than the treatment with individual drugs in inhibiting *T. cruzi* infection.

## Discussion

We have recently shown a main role for the cAMP/Epac1/Rap1b pathway in the establishment of *T. cruzi* infection in different cell lines (Musikant et al., 2017; Ferri et al., 2023). In accordance with these observations, analyses of RNA-seq data obtained after 24 and 48 h of infection revealed that cardiomyocyte infection caused an increase in gene expression of Epac1 and Rap1b, while Rap1GAP2, a protein that induces inactivation of Rap1b, presented a decreased expression (Venturini et al., 2023). In addition, it has been shown that during the chronic phase of the disease, the parasite secretes proteins that could potentiate arrhythmias, presumably by increasing Ca^2+^ intracellular levels. In fact, in cardiomyocytes, Epac-mediated activation is necessary for stimulation of Ca^2+^ induced by Ca^2+^ release through RyR (Oestreich et al., 2007), while Epac1 depletion protects against myocardial I-R damage (Fazal et al., 2017). Interestingly, it has been reported that vitexin, present in *C. oxyacantha*, protects against I-R damage and may act by inhibiting the expression of Epac and Rap1 proteins (Yang et al., 2021). In this context, and considering that the pathway has been successfully targeted in other human pathologies before (Parnell et al., 2015), we evaluated the inhibition of Epac/Rap1b by a natural extract as a potential therapeutic target for CD.

We found that the pre-treatment of the cells with CO-EE or CO-Inf produced a decrease in *T. cruzi* invasion. Moreover, no additive effects were seen when simultaneously pre-treating with CO-EE and the Epac specific inhibitor ESI-09, suggesting that both treatments share the same mechanism of action. Accordingly, CO-EE-treated cells showed a decrease in Rap1b activation, confirming that the extract would inhibit the cAMP/Epac/Rap1b pathway.

Although in our original hypothesis vitexin would be responsible for the inhibition of the Epac/Rap1b pathway, this flavonoid was not identified in the CO-Inf. Molecular studies inferred that solubility of this type of structures is affected by the planarity achieved by rings A, B, and C (Figure 6). A torsion angle is generated between the positions OC2C1’C6’ and the magnitude seems to correlate with solubility (Chebil et al., 2007). Experimental data suggests that when the molecule possesses a C2–C3 double bond at the ring C, the bond angle decreases and its solubility in water declines as well. This could explain why most of the flavonoids, not only vitexin, were detected in CO-EE but not in CO-Inf, although there are other factors to take into consideration such as the degree of glycosylation of the aglycone, the identity and position of the sugar units, or the number of phenols groups in the aromatic rings, which contribute to the highly variable solubility of these compounds.

Interestingly, even when CO-Inf presented a different composition than CO-EE, it was still able to modulate *T. cruzi* infection. Although not conclusive, LC/MS analysis and docking experiments allowed us to hypothesize that several compounds found in both extracts might be mediating the negative modulation on Rap1b activation by potentially inhibiting Epac/Rap1b interaction.

Blind docking simulations on Epac structure showed that vitexin may be interacting with the catalytic domain of Epac with a relatively high score, potentially inhibiting Epac/Rap1b interaction. Other molecules identified in the extracts by LC/MS analysis were also predicted to bind to the Epac/Rap1b interphase with similar, or even higher docking score, than vitexin. Not all compounds were spatially located in the exact pocket than vitexin. Citric acid, caffeic acid, and its esters with threonic acid and glycerol were positioned within the GEF domain, but far from vitexin. However, when sterified with quinic acid, overlapping of at least some portion of the molecule with vitexin was observed. Monomeric and dimeric flavonoids were also situated in a different region compared to vitexin. Still, for the glycosylated version of these flavonoids, at least some superposition was also observed, except for isovitexin. Of the 18 compounds that may bind to the domain, only two were present in CO-Inf but absent in CO-EE. One of them, vicenin-2, is a vitexin-bearing a glucose unit in position C-6 of the apigenin. Remarkably, this compound was placed in the same binding pocket than vitexin, potentially explaining why both extracts share similar effects on the Epac/Rap1 pathway modulation. The other flavonoids present in CO-EE extract, which share similar docking poses and binding scores as vitexin, may elicit similar bioactivity. Although promising, further analysis of the metabolites present in the extracts should be carried out in order to confirm the active compounds and the detailed mechanism of action, and crystal structures must be obtained.

Interestingly, when pre-treating cells with CO-EE and NFX, an increased inhibition of invasion was observed, confirming that both drugs might be acting on different targets. Such a cotreatment would open the possibility of reducing the dosage/time of NFX, which would contribute to avoiding adverse effects. The fact that CO-EE is used to treat cardiac pathologies through its antioxidant and antiarrhythmic properties (Jayalakshmi and Devaraj, 2010; Koch and Malek, 2011; Wang et al., 2013) grants this medicinal herb unique characteristics in the potential treatment of CD, being able to prevent the invasion and spread of *T. cruzi* while treating the damage caused by this parasite in the heart.

Considering that there is a wide variety of therapies that target cAMP-mediated signaling (Parnell et al., 2015), identifying the key components of the cAMP/Epac1/Rap1b pathway could provide an attractive set of new therapeutic targets for the repositioning or development of antiparasitic drugs against CD. In this work, with the long-term goal of getting a more effective, cost-efficient, easy-to-prepare and deliver new treatment for CD, we have described the ability of a natural extract to prevent *T. cruzi* infection *in vitro.*

## Data availability statement

The raw data supporting the conclusions of this article will be made available by the authors, without undue reservation.

## Author contributions

GF: Data curation, Formal analysis, Investigation, Methodology, Validation, Writing – original draft, Writing – review & editing. LF: Data curation, Formal analysis, Investigation, Methodology, Writing – original draft. GM: Data curation, Formal analysis, Investigation, Methodology, Software, Writing – review & editing. DM: Formal analysis, Investigation, Methodology, Writing – review & editing. JP: Conceptualization, Methodology, Resources, Writing – review & editing. ME: Conceptualization, Data curation, Formal analysis, Funding acquisition, Methodology, Project administration, Resources, Supervision, Writing – original draft, Writing – review & editing.
