# Supplementary material for: Host cell cAMP-Epac-Rap1b pathway inhibition by hawthorn extract as a potential target against Trypanosoma cruzi infection
Source: Front Microbiol. 2023 Dec 12;14:1301862. doi: 10.3389/fmicb.2023.1301862 (PMC10754523; doi:10.3389/fmicb.2023.1301862)

S1 Figure. Viability assay.

HELA cells were grown and incubated with 37.5 µM ESI-09, 0.04% CO-EE, 0.4% CO-Inf, simultaneously co-incubated with 0.04% CO-EE and 37.5 µM ESI-09, or respective controls. Cells were then washed and incubated with resazurin for 3 hours. Fluorescence was measured at 590 nm. Results were normalized to control (DMEM only) and expressed as mean ± SD of three or more independent experiments performed in triplicate.

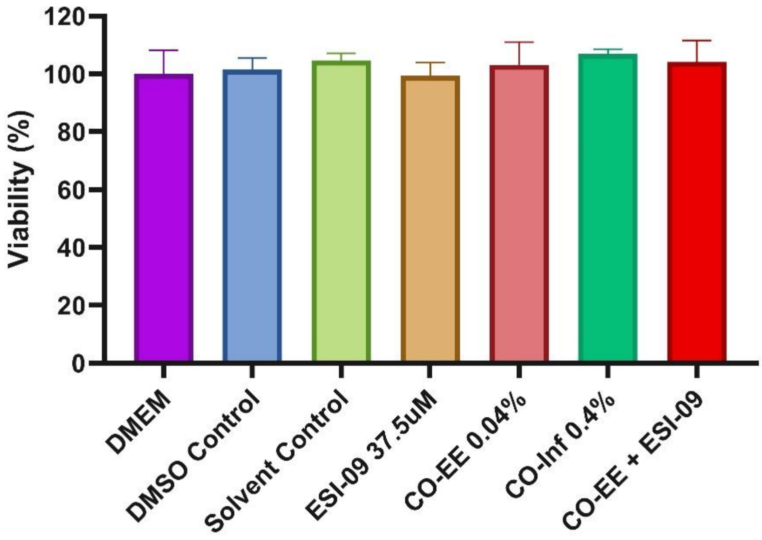

Supplement: Supplementary file 1 [file Data_Sheet_1.PDF]
