# Supplementary material for: Host cell cAMP-Epac-Rap1b pathway inhibition by hawthorn extract as a potential target against Trypanosoma cruzi infection
Source: Front Microbiol. 2023 Dec 12;14:1301862. doi: 10.3389/fmicb.2023.1301862 (PMC10754523; doi:10.3389/fmicb.2023.1301862)
Supplement: Supplementary file 2 [file Data_Sheet_2.PDF]

S2 Figure. Densitometry Analysis of Figure 4

Method: GST Pull-down. 1 ml of GST- or GST-RBD bacterial lysates were spin-mixed with 40 µl of 50% GSH-sepharose at 4°C for 1 h. Then, beads were centrifuged and washed with lysis buffer. Lysates from HA-Rap1-transfected mammalian cells pre-treated for 2 h with 0.04% of CO-EE or solvent control were incubated with RBD-glutathione-agarose resin for 1 h at 4 °C. The resin was washed and eluted with cracking buffer for WB analysis. Image analysis was performed using ImageJ and ImageLab 6.1 software (Bio-Rad). Results are expressed as mean ± SD (n=4) t student test; *** p<0.001, t student test. PD: pull-down.

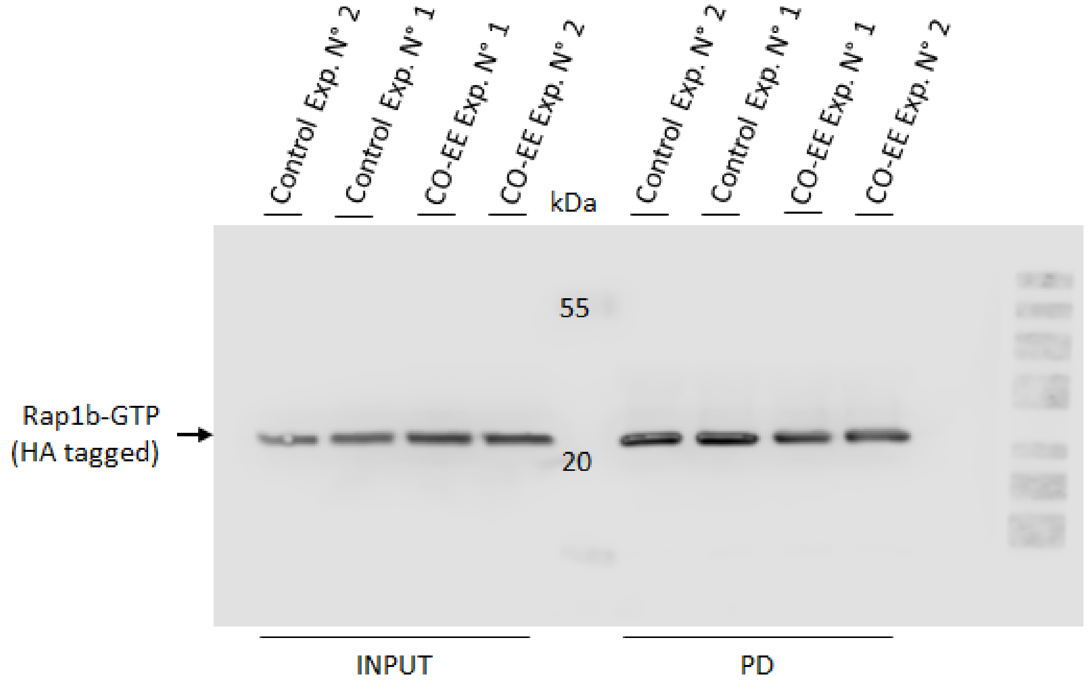

This gel is presented as Figure 4A in the present work.

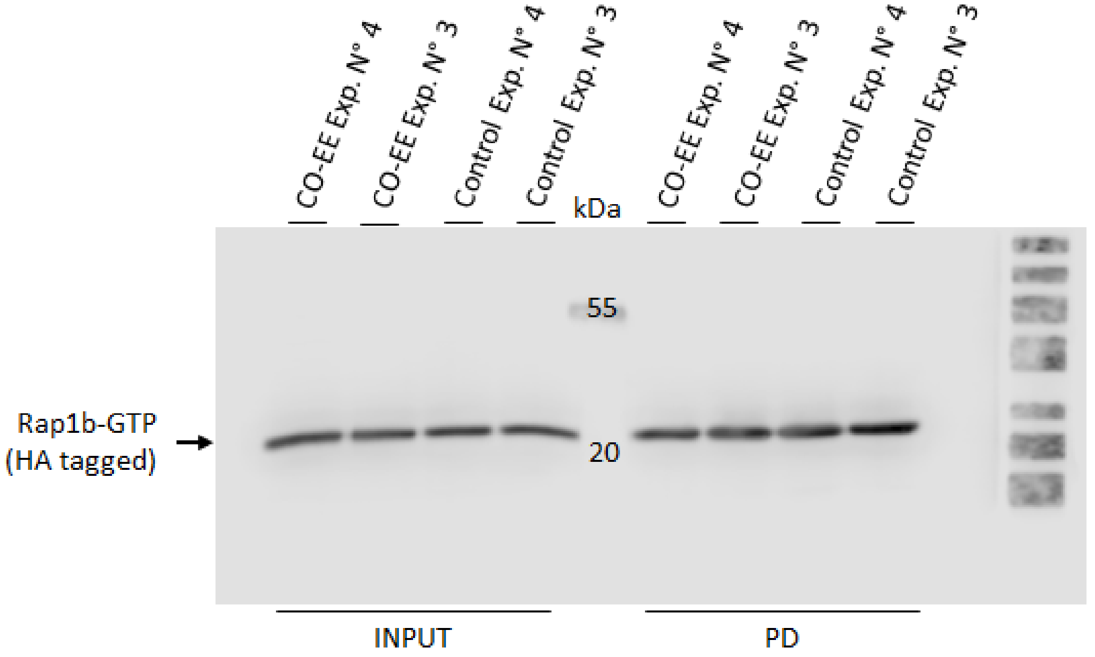

Densitometry analysis

Exp. 1	Lane	**Band intensity**	INPUT/ INPUT Ctrl	PD/ PD Ctrl	**PD/INPUT**	
INPUT Control	4	**30550**	1.00		**Ctrl**	**1.00**
INPUT CO-EE	3	**40080**	1.31		**CO-EE**	**0.59**
PD Control	2	**44666**		1.00		
PD CO-EE	1	**34496**		0.77		
Exp. 2	Lane	**Band intensity**	INPUT/ INPUT Ctrl	PD/ PD Ctrl	**PD/INPUT**	
INPUT Control	4	**18446**	0.60		**Ctrl**	**1.51**
INPUT CO-EE	3	**40250**	1.32		**CO-EE**	**0.61**
PD Control	2	**40744**		0.91		
PD CO-EE	1	**35814**		0.80		
Exp. 3	Lane	**Band intensity**	INPUT/ INPUT Ctrl	PD/ PD Ctrl	**PD/INPUT**	
INPUT Control	3	**38772**	1.27		**Ctrl**	**1.04**
INPUT CO-EE	4	**51678**	1.69		**CO-EE**	**0.68**
PD Control	1	**58752**		1.32		
PD CO-EE	2	**51750**		1.16		
Exp. 4	Lane	**Band intensity**	INPUT/ INPUT Ctrl	PD/ PD Ctrl	**PD/INPUT**	
INPUT Control	3	**45292**	1.48		**Ctrl**	**1.02**
INPUT CO-EE	4	**53766**	1.76		**CO-EE**	**0.55**
PD Control	1	**67570**		1.51		
PD CO-EE	2	**43146**		0.97		

Global analysis

Exp.	1	2	3	4	**Average**	SD
Ctrl	1.00	1.00	1.00	1.00	**1.00**	0.00
CO-EE	0.59	0.40	0.66	0.54	**0.55**	0.11

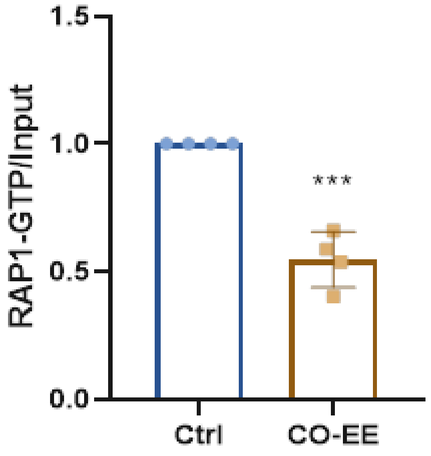

This Figure is presented as Figure 4B in the present work.
