# Supplementary material for: Host cell cAMP-Epac-Rap1b pathway inhibition by hawthorn extract as a potential target against Trypanosoma cruzi infection
Source: Front Microbiol. 2023 Dec 12;14:1301862. doi: 10.3389/fmicb.2023.1301862 (PMC10754523; doi:10.3389/fmicb.2023.1301862)

S3 Figure. Additional Data of Figure 5.

Pre-treated HELA cells (1 h at 0.04% CO-EE or 0.4% CO-Inf or control) were infected with trypomastigotes from *T. cruzi* Y strain (100:1 parasite to cell ratio for 2 h). 48 hs post-infection cells were fixed, stained with DAPI and percentage of invasion determined by fluorescence microscopy. Infection of untreated cells was considered as basal infection. Results are expressed as mean ± SD ($n \geq 3$) *** $p<0.001$, ** $p<0.01$, ANOVA and Dunnett's post-test.

Full quantitative results

Experiment	Control				CO-EE (0.04%)				CO-Inf (0.4%)			
	host nuclei	parasite nuclei	% invasion	% inv.norm.	host nuclei	parasite nuclei	% invasion	% inv.norm.	host nuclei	parasite nuclei	% invasion	% inv.norm.
A1	2051	359	17.50	115.11	1645	74	4.50	29.58	ND	ND	ND	ND
A2	3347	432	12.91	84.89	1349	81	6.00	39.49	1214	67	5.52	36.30
B1	1978	298	15.07	78.94	1067	51	4.78	25.04	1399	66	4.72	24.72
B2	1809	418	23.11	121.06	1956	108	5.52	28.93	1351	83	6.14	32.19
C1	1273	275	21.60	102.29	1171	108	9.22	43.67	2132	229	10.74	50.86
C2	2016	416	20.63	97.71	1643	169	10.29	48.71	1948	147	7.55	35.73
**Average**			**18.47**	**100.00**			**6.72**	**35.90**			**6.93**	**36.02**

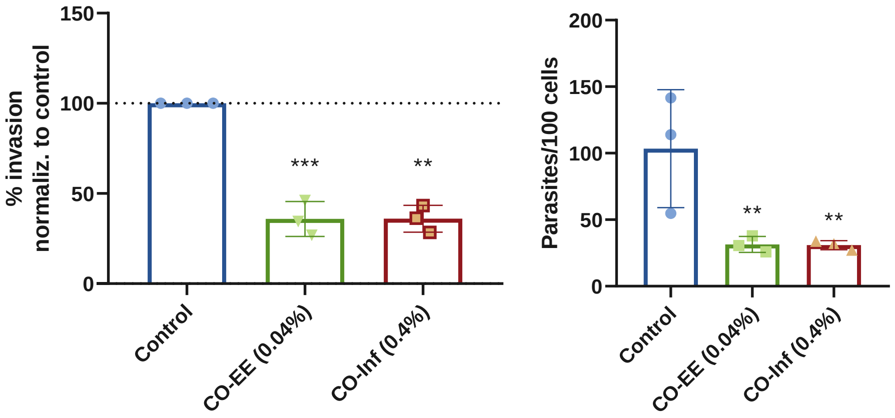

<u>Parasites per infected cell (number of cells counted:500)</u>

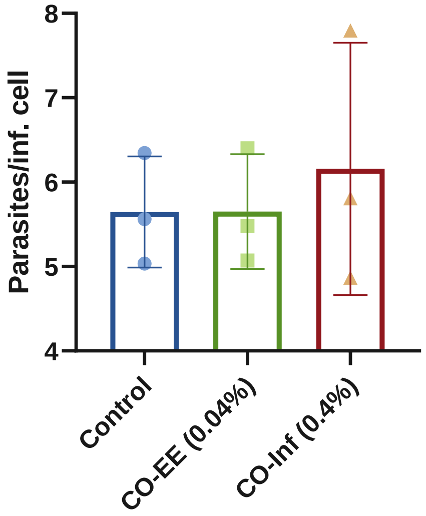

B) Representative images of DAPI staining of infected cells pre-treated with the indicated treatments. CO-EE: hydroalcoholic extract of *C. oxyacantha*. CO-Inf: infusion of the aerial parts of *C. oxyacantha* plant. Scale bar, 5 µm.

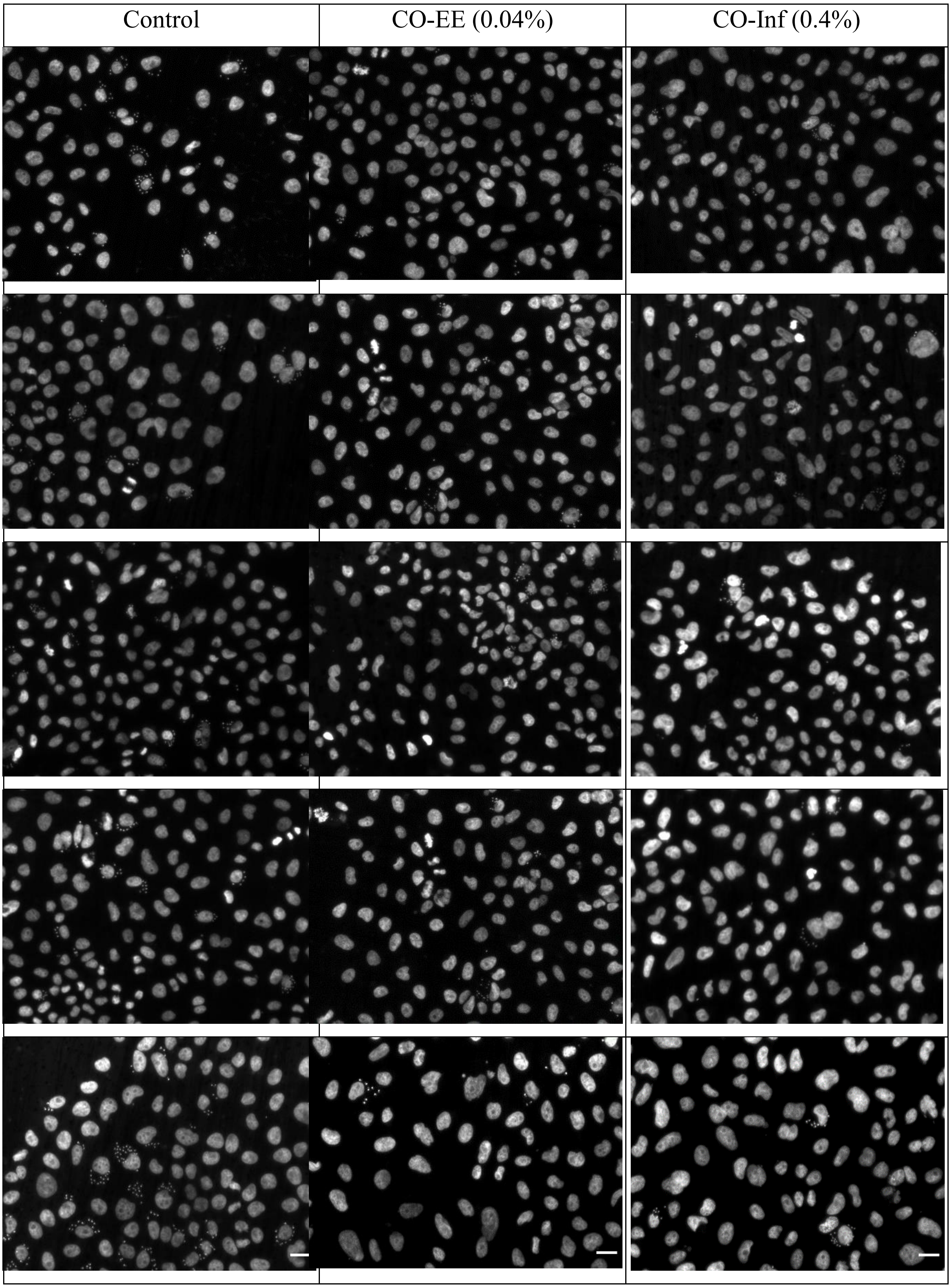

Supplement: Supplementary file 3 [file Data_Sheet_3.PDF]
