# Supplementary material for: Host cell cAMP-Epac-Rap1b pathway inhibition by hawthorn extract as a potential target against Trypanosoma cruzi infection
Source: Front Microbiol. 2023 Dec 12;14:1301862. doi: 10.3389/fmicb.2023.1301862 (PMC10754523; doi:10.3389/fmicb.2023.1301862)

S4 Figure. Additional Data of Figure 9.

Pre-treated HELA cells (1 h at 37.5 µM ESI-09 or 0.04% CO-EE) were infected with trypomastigotes from *T. cruzi* Y strain (100:1 parasite to cell ratio for 2 h). The medium was changed to DMEM medium with different concentrations of NFX or DMSO as control and incubated 48 hs. Then cells were washed, fixed, stained with DAPI and parasites per 100 cells and parasites per infected cells were determined by fluorescence microscopy. Results are expressed as mean ± SD *** $p<0.001$, ** $p<0.01$, * $p<0.1$, ANOVA and Dunnett's post-test.

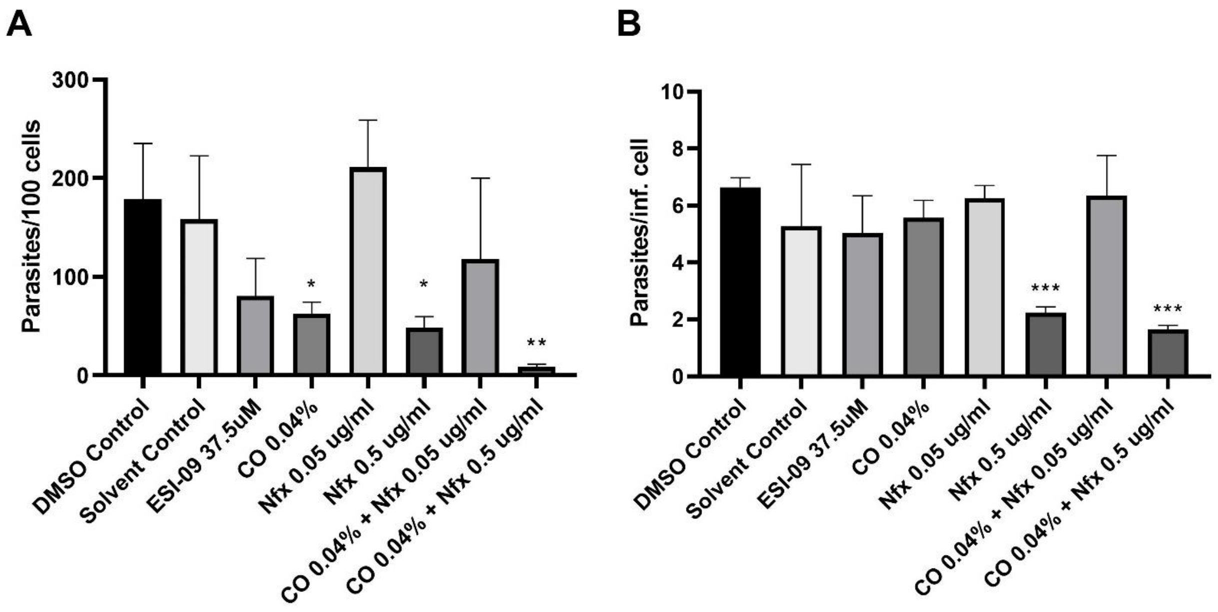

Supplement: Supplementary file 4 [file Data_Sheet_4.PDF]
